# Supplementary material for: Healthcare provider’s adherence to immediate postpartum care guidelines in Gondar province hospitals, northwest Ethiopia: A multicenter study
Source: PLoS One. 2021 Oct 28;16(10):e0259263. doi: 10.1371/journal.pone.0259263 (PMC8553061; doi:10.1371/journal.pone.0259263)
Supplement: S1 Questionnaire — (DOCX) [file pone.0259263.s001.docx]

Annex1: English version of the questionnaire

Part I: Socio-demographic characteristics

| N.O | Questionnaire | Alternative choice for response |
| --- | --- | --- |
| 101 | How old are you? | (In years) |
| 102 | What is your sex? | 1. Male 2. Female |
| 103 | Professional category | 1. Midwifery- Diploma 2. Midwifery-Degree   3. Midwifery-Master’s degree 4. Medical interns   1. IESO 6. Other specify |
| 104 | What is your marital status? | 1. Single 2. Married 3. Divorce 4. Widowed |
| 105 | What is your religion? | 1. Orthodox Christian 2. Muslim 3. Protestant  4. Catholic 5. Other specify………………… |
| 106 | What is your monthly income | (In ETB) |
| 107 | Have you ever read newspapers? | 1. Yes 2. No |
| 108 | Have you ever watched a television? | 1. Yes 2. No |
| 109 | If yes, how often did you watch? | 1. Every day 2. One times per week   3. Two times per week 4. Three and more |
| 110 | Do you have a smartphone or computer | 1. Yes 2. No |
| 111 | Have you ever chew chat? | 1. Yes 2. No |
| 112 | If yes, how often did chew chat per week? |  |
| 113 | Have you ever smoke cigarette? | 1. Yes 2. No |
| 114 | If yes, how often did you smoke cigarette per week? |  |

Part II: **Workplace and profession related questions**

| NO | Questioners | Alternative choices for response |
| --- | --- | --- |
| 201 | Experiences in years (year) |  |
| 202 | Self-rated relation with the nearby boss? | 1. Good 2. Poor |
| 203 | Job satisfaction |  |
|  | Are you satisfied in your payment | 1. Yes 2. No |
|  | Are you satisfied related to promotion | 1. Yes 2. No |
|  | Are satisfied by the supervision process of your organization | 1. Yes 2. No |
|  | Are you satisfied by your coworkers | 1. Yes 2. No |
|  | Are you satisfied by rewards and benefits in your organization | 1. Yes 2. No |
|  | Are you satisfied by the nature of your work | 1. Yes 2. No |
|  | Are you satisfied in your payment | 1. Yes 2. No |
|  | Are you satisfied related to promotion | 1. Yes 2. No |
|  | Are satisfied by the supervision process of your organization | 1. Yes 2. No |
| 204 | Facility type | 1. Primary hospital 2. General hospital  3. Tertiary hospital |
| 205 | Is there an internet connection in your hospital? | 1. Yes 2. No |
| 206 | Is there any guideline for postnatal care/ newborn care in the ward? | 1. Yes 2. No |
| 207 | Have you ever received training on postnatal care? | 1. Yes 2. No |
| 208 | When was the working time (the time during data collection)? | 1. 1. Night 2. Day |
| 209 | Was there any assistance for the delivery? | 1. Yes 2. No |
| 210 | Have you ever received training on essential newborn care? | 1. Yes 2. No |
| 211 | Have you ever received training on BEmONC? | 1. Yes 2.No |
| 212 | If, when did you take the BEmONC training? | 1. Within 2 years 2. Before 2 years |
| 213 | Time taken from home to the health facility |  |
| 214 | Facility location | 1. Urban 2. Semi-urban |
| 215 | Are you intended to stay in the profession for the future? | 1. Yes 2. No |
| 216 | Did the nearby manager regularly monitor for the complete fullness of immediate postnatal cares? | 1. Yes 2. No |
| 217 | Is there workload in the hospital and/or shortage of staffs? | 1. 1. Yes 2. No |
| 218 | Are you interested to work in the delivery unit? | 1. 1. Yes 2. No |
| 219 | Have you ever received training on neonatal resuscitation? | 1. 1. Yes 2. No |
| 220 | If yes, when did you take the training? | - - - 1. Within 2 years 2. Before 2 years |
| 221 | Do you work part-time at a private health facility? | 1. Yes 2. No |
| 222 | Do you have an education to learn while working (like, BSc, MSc by your own)? | 1. Yes 2. No |

**Part VI: Providers adherence related questions on immediate PNC (Observation)**

| S.N | Question | Alternative answer |
| --- | --- | --- |
| 301 | Did the healthcare provider record sex of the neonate? | 1. Yes 2. No |
| 302 | Did the healthcare provider assess APGAR score of the neonate? | 1. Yes 2. No |
| 303 | Did the healthcare provider provide oxytocin? | 1. Yes 2. No |
| 304 | Did the healthcare provider provide Vitamin K for the neonate? | 1. Yes 2. No |
| 305 | Did the healthcare provider measure weight of the neonate? | 1. Yes 2. No |
| 306 | Did the healthcare provider provide TTC eye ointment? | 1. Yes 2. No |
| 307 | Did the healthcare provider massage the uterus? | 1. Yes 2. No |
| 308 | Did the healthcare provider checked maternal vital signs regularly? | 1. Yes 2. No |
| 309 | Did the healthcare provider checked for placental completeness? | 1. Yes 2. No |
| 310 | Did the healthcare provider counsel on danger signs and immunization? | 1. Yes 2. No |
| 311 | Did the healthcare provider checked for uterine contraction? | 1. Yes 2. No |
| 312 | Did the healthcare provider initiate breast feeding within 30 minute to an hour? | 1. Yes 2. No |
| 313 | Did the healthcare provider ensure thermal protection for the neonate? | 1. Yes 2. No |
| 314 | Did the healthcare provider healthcare give advice on breast positioning and attachment? | 1. Yes 2. No |
| 315 | Did the healthcare provider properly document the work, he/she did? | 1. Yes 2. No |
| 316 | Did the healthcare provider measure length of the neonate? | 1. Yes 2. No |
| 317 | Did the healthcare provider measure head circumference of the neonate? | 1. Yes 2. No |
