# Supplementary material for: Healthcare provider’s adherence to immediate postpartum care guidelines in Gondar province hospitals, northwest Ethiopia: A multicenter study
Source: PLoS One. 2021 Oct 28;16(10):e0259263. doi: 10.1371/journal.pone.0259263 (PMC8553061; doi:10.1371/journal.pone.0259263)
Supplement: S1 File — (DOCX) [file pone.0259263.s003.docx]

**Observation and interview guidelines for data collectors (for some specific variables needing clarification)**

1. Newborn babies are highly prone to hypothermia, so keeping them warm is highly recommended. You will tick “Yes” if the birth assistant or the support person thoroughly dries the newborn, change the weight cloth properly, cover the newborn head and initiate skin-to-skin contact.
2. APGAR score should be assessed in the first and fifth minutes after delivery. If the birth attendant of his assistant asses the APGAR score in the first and fifth minutes and takes appropriate actions – will be considered he/she assessed the APGAR score.
3. If the primary birth attendant or the assistant person initiates breastfeeding for the newborn within 30 minutes (depending on the situation within an hour by the WHO recommendation).
4. Counseling on breastfeeding attachment (signs of good attachment including more of the areola are visible above the baby's top lip than below the lower lip; the baby's mouth is wide open; the baby's lower lip is curled outwards; the baby's chin is touching or almost touching the breast) and position (signs of good positioning such as mother in a sitting or lying position, baby facing towards the mother and supported, and baby’s head straight or slightly extended.
5. Presence of a birth assistant means if there is at least one support person during the second stage of labor who has a minimum qualification and can attend labor and managing obstetric complications consider the presence of a birth assistant. The primary role of the assistant during the second stage of labor and the immediate postpartum period will be fetal heart rate auscultation, preparing and arranging necessary materials for delivery and emergency situation, being able to provide essential newborn care, and will do other activities upon request by the primary birth attendant.
6. Oxytocin should be given intramuscularly immediately after delivery of the last baby in case of higher-order pregnancy. If the healthcare provider or the assistant gives oxytocin to the women within a minute for single tone pregnancy or after the delivery of the last baby in case multiple pregnancies, will be considered oxytocin is given.
7. After giving oxytocin and delivering the placenta, it is recommended to massage the fundal part of the uterus until it gets contracted. This will ensure the presence or absence of uterine atony.
8. After placenta delivery, it is a must to routinely examine the placenta for completeness by visualizing the lobes and lifting the cord, and holding the placenta up, so both layers of membranes can be inspected for completeness. If the primary birth assistant or the support person did the aforementioned thins tick a “yes”.
9. Documentation is an important monitoring and evaluation tool to look for clear descriptions about certain activities in detail. Thus, you data collectors are expected to fill “Yes” for the question “did the healthcare provider properly document, if he/she writes the outcome of the pregnancy (stillbirth, alive, singleton/twin APGAR score, Weight, placental completeness etc.), presence or absence of complications and managements done, medications given or prescribed (oxytocin or other), treatment or follow up plans, and name of healthcare providers involved (including signature).
