# Supplementary material for: Healthcare provider’s adherence to immediate postpartum care guidelines in Gondar province hospitals, northwest Ethiopia: A multicenter study
Source: PLoS One. 2021 Oct 28;16(10):e0259263. doi: 10.1371/journal.pone.0259263 (PMC8553061; doi:10.1371/journal.pone.0259263)
Supplement: Attachment — Submitted filename: PONE-D-21-13480_reviewer.pdf [file pone.0259263.s004.pdf]

PLOS ONE

Healthcare provider’s adherence to immediate postpartum care guidelines in Gondar province hospitals, northwest Ethiopia: A multicenter study

--Manuscript Draft--

**Manuscript Number:**	PONE-D-21-13480
**Article Type:**	Research Article
**Full Title:**	Healthcare provider’s adherence to immediate postpartum care guidelines in Gondar province hospitals, northwest Ethiopia: A multicenter study
**Short Title:**	Healthcare provider’s adherence to immediate postpartum care guidelines in Gondar province hospitals
**Corresponding Author:**	Azmeraw Ambachew Kebede, MSc<br>University of Gondar College of Medicine and Health Sciences<br>Gondar, Amhara regional State ETHIOPIA
**Keywords:**	Adherence; Ethiopia; healthcare providers; immediate postpartum care
**Abstract:**	Background<br><br>The immediate postpartum period is the most critical time for both the mother and the newborn. However, it is the most neglected part of the maternal continuum of care and evidence in this regard was scarce in Ethiopia. Therefore, this study aimed to assess the healthcare provider’s adherence to immediate postpartum care guidelines and associated factors in hospitals of Gondar province.<br><br>Methods<br><br>A multicenter observational cross-sectional study was conducted among 406 healthcare providers from 15 th November 2020 to 10 th March 2021. Data were collected through face-to-face interviews and direct observation using a structured questionnaire and standardized checklist respectively. Data was entered into EPI INFO 7.1.2 and analyzed by SPSS version 25. Both bivariable and multivariable logistic regression analyses were carried out. The level of significance was declared based on the adjusted odds ratio (AOR) with a 95% confidence interval (CI) at a p-value of ≤ 0.05.<br><br>Results<br><br>Overall, 42.4% (95% CI: 37.5, 47.2) of healthcare providers had complete adherence to immediate postpartum care guidelines. Having birth assistant (AOR = 1.87; 95% CI: 1.10, 9.67), being married (AOR= 1.59; 95% CI: 1.15, 3.31), availability of postpartum care guidelines at the maternity ward (AOR= 2.39; 95% CI: 1.44, 3.98), received basic emergency obstetric and newborn care (BEmONC) training (AOR= 2.1; 95% CI: 1.2, 3.6), and monthly income of ≥ 10001 Ethiopian birr (AOR= 3.55; 95% CI: 1.30, 9.67) were positively associated with healthcare providers adherence to immediate postpartum care guidelines.<br><br>Conclusion<br><br>This study indicated that health worker’s adherence to immediate postpartum care guidelines was low. Hiring adequate health workers, availing postpartum guidelines at the maternity ward, improving the salary of healthcare workers, and provision of BEmONC training will have a great role in improving healthcare provider’s adherence to immediate postpartum care guidelines.
**Order of Authors:**	Azmeraw Ambachew Kebede, MSc
	Birhan Tsegaw Taye
	Kindu Yinges Wondie

Powered by Editorial Manager® and ProduXion Manager® from Aries Systems Corporation

	Agumas Eskezia Tiguh
	Getachew Azeze Eriku
	Muhabaw shumye Mihret
**Additional Information:**	
**Question**	**Response**
**Financial Disclosure**<br><br>Enter a financial disclosure statement that describes the sources of funding for the work included in this submission. Review the submission guidelines for detailed requirements. View published research articles from *PLOS ONE* for specific examples.<br><br>This statement is required for submission and **will appear in the published article** if the submission is accepted. Please make sure it is accurate.<br><br>**Unfunded studies**<br>Enter: *The author(s) received no specific funding for this work.*<br><br>**Funded studies**<br>Enter a statement with the following details:<br>• Initials of the authors who received each award<br>• Grant numbers awarded to each author<br>• The full name of each funder<br>• URL of each funder website<br>• Did the sponsors or funders play any role in the study design, data collection and analysis, decision to publish, or preparation of the manuscript?<br>• **NO** - Include this sentence at the end of your statement: *The funders had no role in study design, data collection and analysis, decision to publish, or preparation of the manuscript.*<br>• **YES** - Specify the role(s) played.<br><br>* typeset	The author(s) received no specific funding for this work.
**Competing Interests**<br><br>Use the instructions below to enter a competing interest statement for this submission. On behalf of all authors, disclose any competing interests that could be perceived to bias this	he authors have declared that no competing interests exist

Powered by Editorial Manager® and ProduXion Manager® from Aries Systems Corporation

work—acknowledging all financial support and any other relevant financial or non-financial competing interests.<br><br>This statement is required for submission and **will appear in the published article** if the submission is accepted. Please make sure it is accurate and that any funding sources listed in your Funding Information later in the submission form are also declared in your Financial Disclosure statement.<br><br>View published research articles from *PLOS ONE* for specific examples.<br><br>**NO authors have competing interests**<br><br>Enter: *The authors have declared that no competing interests exist.*<br><br>**Authors with competing interests**<br><br>Enter competing interest details beginning with this statement:<br><br>*I have read the journal's policy and the authors of this manuscript have the following competing interests: [insert competing interests here]*<br><br>* typeset	
**Ethics Statement**<br><br>Enter an ethics statement for this submission. This statement is required if the study involved:<br><br>• Human participants<br>• Human specimens or tissue<br>• Vertebrate animals or cephalopods<br>• Vertebrate embryos or tissues<br>• Field research<br><br>Write "N/A" if the submission does not require an ethics statement.	The study was conducted under the Ethiopian Health Research Ethics Guideline and the declaration of Helsinki. Ethical clearance was obtained from the Institutional Ethical Review Board (IRB) of the University of Gondar (Reference number: V/P/RCS/05/413/2020). A formal letter of administrative approval was gained from each selected hospital. Written informed consent was taken from each of the study participants after a clear explanation of the aim of the study

Powered by Editorial Manager® and ProduXion Manager® from Aries Systems Corporation

General guidance is provided below. Consult the submission guidelines for detailed instructions. **Make sure that all information entered here is included in the Methods section of the manuscript.**<br><br>**Format for specific study types**<br><br>**Human Subject Research (involving human participants and/or tissue)**<br>• Give the name of the institutional review board or ethics committee that approved the study<br>• Include the approval number and/or a statement indicating approval of this research<br>• Indicate the form of consent obtained (written/oral) or the reason that consent was not obtained (e.g. the data were analyzed anonymously)<br><br>**Animal Research (involving vertebrate animals, embryos or tissues)**<br>• Provide the name of the Institutional Animal Care and Use Committee (IACUC) or other relevant ethics board that reviewed the study protocol, and indicate whether they approved this research or granted a formal waiver of ethical approval<br>• Include an approval number if one was obtained<br>• If the study involved *non-human primates*, add *additional details* about animal welfare and steps taken to ameliorate suffering<br>• If anesthesia, euthanasia, or any kind of animal sacrifice is part of the study, include briefly which substances and/or methods were applied<br><br>**Field Research**<br><br>Include the following details if this study involves the collection of plant, animal, or other materials from a natural setting:<br>• Field permit number<br>• Name of the institution or relevant body that granted permission	
**Data Availability**<br><br>Authors are required to make all data underlying the findings described fully	Yes - all data are fully available without restriction

available, without restriction, and from the time of publication. PLOS allows rare exceptions to address legal and ethical concerns. See the PLOS Data Policy and FAQ for detailed information.<br><br>A Data Availability Statement describing where the data can be found is required at submission. Your answers to this question constitute the Data Availability Statement and **will be published in the article**, if accepted.<br><br>**Important:** Stating 'data available on request from the author' is not sufficient. If your data are only available upon request, select 'No' for the first question and explain your exceptional situation in the text box.<br><br>Do the authors confirm that all data underlying the findings described in their manuscript are fully available without restriction?	
**Describe where the data may be found in full sentences. If you are copying our sample text, replace any instances of XXX with the appropriate details.**<br><br>• If the data are **held or will be held in a public repository**, include URLs, accession numbers or DOIs. If this information will only be available after acceptance, indicate this by ticking the box below. For example: *All XXX files are available from the XXX database (accession number(s) XXX, XXX.*).<br>• If the data are all contained **within the manuscript and/or Supporting Information files**, enter the following: *All relevant data are within the manuscript and its Supporting Information files.*<br>• If neither of these applies but you are able to provide **details of access elsewhere**, with or without limitations, please do so. For example:<br><br>*Data cannot be shared publicly because of [XXX]. Data are available from the XXX Institutional Data Access / Ethics*	All relevant data are within the manuscript and its Supporting Information files.

Committee (contact via XXX) for researchers who meet the criteria for access to confidential data.<br><br>*The data underlying the results presented in the study are available from (include the name of the third party and contact information or URL).*<br>• This text is appropriate if the data are owned by a third party and authors do not have permission to share the data.<br><br>* typeset	
Additional data availability information:	Tick here if the URLs/accession numbers/DOIs will be available only after acceptance of the manuscript for publication so that we can ensure their inclusion before publication.

Powered by Editorial Manager® and ProduXion Manager® from Aries Systems Corporation

Click here to access/download;Manuscript;Manuscript on immediate PPP.docx

Research article

Healthcare provider's adherence to immediate postpartum care guidelines in Gondar province hospitals, northwest Ethiopia: A multicenter study

Azmeraw Ambachew Kebede[1*], Birhan Tsegaw Taye [2], Kindu Yinges Wondie[1], Agumas Eskezia Tiguh[1], Getachew Azeze Eriku[3], Muhabaw Shumye Mihret[1]

[1] Department of Clinical Midwifery, School of Midwifery, College of Medicine and Health Sciences, University of Gondar, Gondar Ethiopia; [2]Department of Clinical Midwifery, College of Medicine and Health Sciences, Debre Berhan University, Debre Berhan Ethiopia;[3] Department of physiotherapy, College of Medicine and Health Sciences, University of Gondar, Gondar Ethiopia .

*azmuzwagholic@gmail.com (AAK)

Abstract

Background

The immediate postpartum period is the most critical time for both the mother and the newborn. However, it is the most neglected part of the maternal continuum of care and evidence in this regard was scarce in Ethiopia. Therefore, this study aimed to assess the healthcare provider's adherence to immediate postpartum care guidelines and associated factors in hospitals of Gondar province.

Methods

A multicenter observational cross-sectional study was conducted among 406 healthcare providers from 15th November 2020 to 10th March 2021. Data were collected through face-to-face interviews and direct observation using a structured questionnaire and standardized checklist respectively. Data was entered into EPI INFO 7.1.2 and analyzed by SPSS version 25. Both bivariable and multivariable logistic regression analyses were carried out. The level of significance was declared based on the adjusted odds ratio (AOR) with a 95 % confidence interval (CI) at a p-value of ≤ 0.05.

Results

Overall, 42.4% (95% CI: 37.5, 47.2) of healthcare providers had complete adherence to immediate postpartum care guidelines. Having birth assistant (AOR = 1.87; 95% CI: 1.10, 9.67), being married (AOR= 1.59; 95% CI: 1.15, 3.31), availability of postpartum care guidelines at the maternity ward (AOR= 2.39; 95% CI: 1.44, 3.98), received basic emergency obstetric and newborn care (BEmONC) training (AOR= 2.1; 95% CI: 1.2, 3.6), and monthly income of ≥ 10001 Ethiopian birr (AOR= 3.55; 95% CI: 1.30, 9.67) were positively associated with healthcare providers adherence to immediate postpartum care guidelines.

Conclusion

This study indicated that health worker's adherence to immediate postpartum care guidelines was low. Hiring adequate health workers, availing postpartum guidelines at the maternity ward, improving the salary of healthcare workers, and provision of BEmONC training will

have a great role in improving healthcare provider's adherence to immediate postpartum care guidelines.

Keywords: Adherence, Ethiopia, healthcare providers, immediate postpartum care

Introduction

The immediate postpartum period (IPPP) is the first 24 hours after delivery [1]. The IPPP is very dangerous for both the mother and the newborn [2]. This time in particular will determine the long-term health and well-being of neonates [3,4]. However, this IPPP is neglected by many health professionals and is rarely given attention [1].

In 2017, the global maternal mortality (MM) was about 295,000 (an estimate of 211 maternal deaths per 100,000 live births) [5] and 2.5 million neonatal mortality [6]. Of these, 94 percent of all maternal and 80% neonatal deaths occur in developing countries [6,7]. Sub-Saharan Africa (SSA) merely accounted for more than two-thirds of the global MM (i.e., 542 maternal deaths per 100, 000 live births) [5]. Thus, about 20 to 44% of these maternal deaths occur during the PPP [8]. This can be reversed by providing appropriate and timely postnatal care (PNC) by qualified health professionals [4]. This is because delays in getting quality immediate PNC services are one of the main causes of MM [9].

Sub-Saharan Africa has been faced with many challenges to safeguard the optimal health of both the mother and newborn [10]. One of the reasons is inequitable and discriminatory maternal health service provision based on the specific status of women [11]. Ethiopia is one of the SSA countries with the highest maternal and neonatal mortalities and low healthcare access. According to the 2017 fragile states index, Ethiopia was one of the fifteenth countries grouped under the "very high alert" category [5].

Although maternal and neonatal mortality in Ethiopia has been declining since 2000, the reduction is still unsatisfactory. Thus, the MM was 871 in 2000,(12) 673 in 2005,(13) 676 in 2011 [14] and 412 per 100,000 live births in 2016 [15]. Besides, neonatal mortality (NM) has fallen from 49 in 2000 to 39 in 2005,(12,13) 37 in 2011 to 29 per 1000 live births in 2016 [14,15]. In Ethiopia as a country, a lot has been done to increase maternal healthcare service utilization with the promise of reducing the maternal mortality ratio from 412 to 199 per 100,000 live births and the neonatal mortality rate from 29 to 10 per 1000 live births by 2020

[16]. However, it is impossible to achieve this goal without the healthcare provider's adherence to maternal and newborn guidelines.

Moreover, the failure of healthcare providers to comply with immediate postpartum care guidelines (IPPCG) and postpartum counseling contributes to poor PNC uptake. Evidence supports that the right steps taken by health professionals can greatly reduce MM, principally during the IPPP [17]. To reduce maternal and neonatal mortalities on a permanent and sustainable basis, healthcare professionals, especially midwives, play the lion's share [18] as healthcare providers, in specific midwives, are the frontline care providers for women and newborns [19]. Empirical evidence showcases that an estimated 41% of maternal and 39% of neonatal deaths can be prevented by expanding midwifery-delivered interventions [18]. In addition, ensuring quality midwifery care is a prerequisite for achieving the sustainable development goals (SDG), in particular, targets 3.1 and 3.2 [20,21].

Studies examining the healthcare provider's adherence to IPPCG are scarce. Most of the previously published literature focuses on women's utilization of PNC and associated factors. Only one study has been conducted in Ethiopia regarding healthcare provider's adherence to IPPCG. According to this study, 22.8% of healthcare providers had complete adherence to IPPCG [22]. But, while the study intended to assess healthcare provider's adherence to IPPCG, the data were collected from the women. It fails to assess which specific factors will affect the adherence level of healthcare providers to the existing IPPCG. Therefore, this is one of the first studies to ascertain the healthcare provider's adherence to IPPCG as to the authors' deep review. Also, this study aimed to identify individual, workplace and profession-related factors associated with healthcare provider's adherence to the IPPCG in Gondar province hospitals, northwest Ethiopia.

Methods and materials

Study design, area, and period

A multicenter institution-based cross-sectional study was conducted from 15th November 2020 to 10th March 2021. The study was conducted at Hospitals in Gondar province Amhara national regional state, northwest Ethiopia. In the Gondar province, there are four zones, namely South Gondar, Central Gondar, West Gondar, and North Gondar zones. There are a total of 22 hospitals in Gondar province. Among these, the University of Gondar and Debre Tabor's are referral hospitals. The remaining 20 hospitals are primary hospitals except Debark general hospital. These hospitals are serving for about more than 10 million population in the four zones of Gondar province and surrounding zones like North Wollo and Waghimra zone.

Study population

All healthcare providers working at the maternity ward and attending women at or after 28 weeks of gestation (i.e. after fetal viability in Ethiopian context) were the study population. These include medical doctors, midwives, and integrated emergency surgeon officers (IESO) working at hospitals in northwest Ethiopia during the data collection period were included.

Sample size determination and sampling procedure

The sample size for this study was determined by using a single population proportion formula by considering the following assumptions: 95% level of confidence, 50% provider's adherence to immediate postpartum care guidelines, and 5% margin of error.

$$n = \frac{Z\alpha/2)^2 p(1-p)}{d^2} = n = \frac{(1.96)^{2*}0.5\ (1-0.5)}{(0.05)2} = 384$$

Where, n = required sample size, α = level of significant, z = standard normal distribution curve value for 95% confidence level = 1.96, p = providers adherence to immediate postnatal care guidelines, d = margin of error. After considering a non-response rate of 10%, we obtained a total sample size of 422. Data were collected from 15 hospitals (2 tertiary hospitals, 1 general hospital, and 12 primary hospitals). The seven primary hospitals were excluded due to their very low monthly delivery report. The lists of healthcare providers

were obtained from each hospital and the sampling frame was designed by numbering the list of healthcare providers. Then, the total sample size was distributed to each selected hospital proportionally. Finally, the participants were selected by a simple random sampling technique using a table of random generation.

Variables of the study

The dependent variable for this study was adherence to IPPCG whereas the independent variables were age, sex, educational level, marital status, availability of smartphone or computer, and availability of media, year of experience, relation to the nearby boss, intention to stay in the profession, job satisfaction, facility type, availability of internet, availability of postnatal guideline in the ward, working time, training on essential newborn care, training on basic emergency obstetric and newborn care (BEmONC), presence of regular follow-up by the manager, workload or shortage of staff in the delivery room, and location of the health facility.

Operational definitions and measurements

Immediate postpartum care: a total of 17 checklists or questions were prepared to assess healthcare providers adhere to the IPPCG. Each checklist has a "yes "or "no" response giving a score of 0-17 (i.e., a score of 1 was given for "yes" and 0 for "no" response). Likewise, the healthcare provider's adherence to IPPCG was dichotomized as complete adherence if the healthcare provider performs all the offered checklists (which was coded as "1") and incomplete adherence if the provider fails to perform all the offered checklists (which was coded as ‘‘0’’). The immediate postpartum period is 6 hours after delivery in the Ethiopian context [22].

Data collection tools, methods, and procedures

The data collection tool was developed by reviewing literature and guidelines [22,23] and data were collected using a structured questionnaire and checklists through face-to-face interviews and direct observation respectively. The questionnaire was assessed by a group of researchers to evaluate and enhance the items in the question. The questionnaire contains socio-demographic characteristics, professional and environment-related factors, and

questions assessing the provider's adherence to IPPCG. About 20 personnel recruited for the data collection process. These include 15 Diploma midwives for data collection and 5 BSc midwives for supervision.

Data quality control

Before the actual data collection, a pretest was done on 5% of healthcare providers outside of the study area. The data collectors and supervisors were trained about the interview technique and overall data collection process for 3 days. During data collection, the questionnaire was checked for completeness by the supervisors.

Data processing and analysis

Data were checked and coded manually. EPI INFO version 7.1.2 and SPSS version 25 were used for data entry and analysis respectively. Descriptive statistics were used to present participants' characteristics and adherence to the IPPCG. The binary logistic regression model was fitted and bivariable and multivariable logistic regression analyses were undertaken. Variables having a p-value of less than 0.2 at a bivariable logistic regression analysis were candidates for multivariable logistic regression analysis. In the multivariable logistic regression, the level of significance was claimed based on AOR with 95% CI at a p-value of ≤ 0.05.

Ethics approval and consent to participate

The study was conducted under the Ethiopian Health Research Ethics Guideline and the declaration of Helsinki. Ethical clearance was obtained from the Institutional Ethical Review Board (IRB) of the University of Gondar (**Reference number: V/P/RCS/05/413/2020**). A formal letter of administrative approval was gained from each selected hospital. Written informed consent was taken from each of the study participants after a clear explanation of the aim of the study.

Results

Socio-demographic characteristics

In this study, a total of 422 healthcare providers were involved, giving a response rate of 96.2%. The mean age of the study participants was 28.4 years (SD ±4.7) and 60.8% of the participants were between the age group of 26 to 30 years. The majority of the healthcare providers were Degree midwives with an average monthly income of 5861.3 Ethiopian Birr (ETB) (**Table 1**).

Workplace and profession-related characteristics

Of the total study participants, more than three-fourths were satisfied with their profession and 71.4% of the healthcare providers had an intention to stay in their profession. About 31% and 38.4% of the participants received BEmONC and essential newborn care training. Eighty-three percent of the healthcare providers had a good interest to work in the delivery room and 77.3% of the participants had good relations with the nearby manager (**Table 2**)

Healthcare providers' adherence to immediate postpartum care guidelines

The proportion of healthcare provides adhering to IPPCG was 42.4% (95% CI: 37.5, 47.2). Tetracycline eye ointment application (96.6%), oxytocin administration (95.6%), and measuring the weight of the newborns (95.1%) were areas of good practice. The level of adherence to IPPCG varied across individuals (**Table 3**).

Factors associated with adherence to immediate postpartum care guidelines

Multivariable logistic regression analysis revealed that marital status of healthcare providers, presence of postpartum care guidelines in the maternity ward, presence of an assistant for the delivery, received training on BEmONC, and average monthly income were factors significantly associated with healthcare provider's adherence to IPPCG.

Healthcare providers who got marriage were 1.59 times more likely to completely adhere to IPPCG as compared to their counterparts (AOR= 1.59; 95% CI: 1.15, 3.31). Likewise, healthcare providers who had an assistant for delivery were 1.87 times more likely to have

had complete adherence to IPPCG as compared to those healthcare providers who hadn't have a birth assistant for the delivery (AOR= 1.87; 95% CI: 1.10, 9.67).

The odds of adhering to IPPCG were 2.39 times higher among healthcare workers who had postpartum care guidelines in their hospital than their counterparts (AOR= 2.39; 95% CI: 1.44, 3.98). Similarly, the health care providers who received BEmONC training were 2.1 times more likely to adhere to IPPCG as compared to the reference group (AOR= 2.1; 95% CI: 1.2, 3.6). Moreover, the likelihood of adhering to IPPCG was 3.55 times higher among healthcare providers earning ≥10001 ETB than those professionals with lesser monthly income (AOR= 3.55; 95% CI: 1.30, 9.67) (**Table 4**).

Discussion

Healthcare providers are strongly encouraged to adhere to IPPCG so that any complications on the neonates and mothers will be recognized and managed in a timely manner. These timely problem identifications and interventions could then reduce maternal and neonatal mortality occurring during the IPPP on a sustainable basis. Hence, the current study aimed at assessing healthcare provider's adherence to IPPCG and associated factors in hospitals Gondar province, northwest Ethiopia. Accordingly, the proportion of healthcare providers adhering to IPPCG was 42.4% and independently predicted by the availability of birth assistants, married marital status, availability of postpartum care guidelines at the maternity ward, provision of BEmONC training, and monthly income of ≥ 10001 ETB.

In the present study, about two-fifth (42.4%) of healthcare providers adhered to IPPCG. This finding is higher than a study conducted in Mekelle, Ethiopia_22.8% [22]. The discrepancy might be due to variation in the study population. The study from Mekelle includes nurses and midwives whereas our study incorporates midwives, medical doctors, and IESO. Previously nurse professionals take over the maternal and neonatal unit. Nowadays, however, midwives along with medical doctors are more available in the maternity wards and it is known that midwives are more close to maternal health [24,25]. Besides, the higher proportion may be ascribed to maternal and neonatal health is a major public health issue and getting attention worldwide. Hence, training is being given regularly for healthcare providers regarding maternal and neonatal health so as to increasing their knowledge, skill, and self-

efficacy [26]. In addition to increasing their knowledge and skill, training might be a source of income and used as entertainment and recreation for providers. The other most important contributing factor might be the Ethiopian Midwife Association (EMwA) is doing an incredible effort for midwives such as continuing professional development across the country. Ethiopian midwife association in collaboration with the Ethiopian ministry of health and other non-governmental organization are doing many activities including giving training for healthcare providers and doing researches which further enhances the grow up of midwifery profession [22,28].

The adherence level of healthcare workers towards tetracycline eye ointment application, oxytocin provision, and weight measurement was 96.6%, 95.6%, and 95.1% respectively. The result of this study is higher compared to the findings from Mekelle in which healthcare provider's adherence to tetracycline application_67.7%, oxytocin administration_85.1%, and weight measurement_91.1% [22]. The higher proportion of healthcare provider's adherence to IPPCG in this study might be due to the time gap. In addition, workplace, and profession-related factors like training, job satisfaction, and relation with the nearby manager and interest to work in the delivery room may contribute to the variation. In this study, about 38.4%, 31%, and 71.4% of healthcare providers received training on essential newborn care, BEmONC, and intended to stay in the profession respectively. Empirical evidence indicated that training regarding maternal and neonatal health is very crucial and help professional to comply with the available standards [26,29]. In the current study, the healthcare provider's adherence to neonatal length and head circumference measurements were found to be low which was 48.8% and 49% respectively. This might be due to the perception that length and head circumference are not acute and determinant for the newborn. In this regard, we strongly recommend healthcare providers to perform all components of immediate postpartum care standards. It then helps to decrease maternal and neonatal mortality and morbidity that occur during the PP.

The present study revealed that the odds of having complete adherence to IPPCG were 2.1 times higher among healthcare providers who have received BEmONC training than healthcare workers who hadn't received BEmONC training. This is due to the fact that BEmONC training is constantly provided to trainees with the aim of fostering basic knowledge and skills on the management of emergency obstetric and neonatal health

complications. Therefore, health professionals receiving BEmONC training are likely to have more knowledge and skills about postpartum services thereby comply with the respective guidelines as compared to non-trained health professionals. In connection to this, existing evidence support that obstetric care providers who received in-service training are more likely to know and practice essential newborn care satisfactorily [30]. Moreover, BEmONC training is essential to combat unacceptably high maternal and neonatal mortalities, particularly in developing countries [29]. This suggests the need for providing BEmONC training for healthcare providers working at maternity and neonatal units regularly thereby moving forward to achieve the SDGs.

The availability of postpartum care guidelines at the maternity ward is an important determinant factor for healthcare provider’s complete adherence to IPPCG. Healthcare providers who had an access to IPPCG at the hospital were 2.39 times more likely to adhere to IPPCG compared with those healthcare providers who haven’t accessed the protocol. This might be due to the logic that if instructions are placed in the delivery room in the form of a document or posted on the wall, health professionals can then easily oversee frequently, recall and demonstrate each step proficiently over time. Along with training, IPCG guidelines should be given to health institutions and individual healthcare providers in the form of soft copy or printed material to enhance retention of knowledge and skill.

The odds of having complete adherence to IPPCG among married healthcare providers were 1.59 times higher as compared to those who were not married. This can be possibly explained by married health professionals will take duties and can act responsibly for everything. Evidence indicated that married healthcare providers have a positive attitude towards reproductive health services compared with unmarried individuals [31]. Likewise, this study found that healthcare providers who had an assistant for the delivery were 1.87 times more likely to have had complete adherence to the immediate postpartum care guidelines as compared to those healthcare providers who hadn’t have a birth assistant for the delivery. This is because if there is no assistant during childbirth, the providers may not be able to follow the guidelines properly due to workload or may ignore it altogether. In this regard, allocating sufficient skilled birth attendants at the maternity ward would help provider’s adherence to guidelines. Because it curtails task overload and facilitates teamwork, thus early recognition and treatment of complications in case of emergency.

Lastly, the current study determined that healthcare providers having a monthly income of greater than 10001 ETB were 3.55 times more likely to perform the postpartum care guidelines properly as compared to healthcare providers who had a monthly income of less than 5000 ETB. This is possibly due to the fact that a balanced salary inspires healthcare provider's not only to love their profession but also encourage them to do all the work properly [32]. Unable to get appropriate payment for their work may cause a hopeless future and overall dissatisfaction to healthcare providers. Hence, health policymakers and other stalk holders need to give special consideration to benefit packages of healthcare providers at different levels of the healthcare system.

Limitations of the study

The cross-sectional nature of the study design may not possible to generalize the causal relationship between healthcare provider's adherence to IPPG and the hypothesized predictor variables. Besides, we didn't find enough literature to compare our results with others and make it difficult to discuss in detail. Despite these limitations, the finding of this study provides valuable information regarding healthcare provider's adherence to IPPG.

Conclusion

The healthcare provider's adherence to IPPCG was found to be low. Having birth assistants, being married, availability of postpartum care guidelines at the maternity ward, received BEmONC training, and higher monthly income was positively associated with healthcare provider's adherence to IPPCG. Thus, the federal ministry of health and the regional health bureau should pay a special emphasis on hiring adequate health professionals in the maternity ward, constant provision of training, and praiseworthy payments for healthcare providers.

Abbreviations

AOR, adjusted odds ratio; BEmONC, basic emergency obstetric and newborn care; CI, confidence interval; COR, crude odds ratio; ETB, Ethiopian birr; IPPCG, immediate postpartum care guidelines; IPPP, immediate postpartum period; MM, maternal mortality; PNC, postnatal care; SPSS, statistical package for social science; SSA, Sub-Saharan Africa

Consent to publish: Not applicable

Availability of data and materials: The datasets used and/or analyzed during the current study are available from the corresponding author on reasonable request.

Competing interests: The authors declare that they have no competing interests.

Funding: The research was not supported by any grant

Acknowledgments

We would like to thank the University of Gondar for providing study ethical clearance to conduct this study. Our gratitude also goes to all data collectors and study participants. We are glad to Hospitals in Gondar province for writing permission letter.

Authors' Contributions

Conceptualization: Azmeraw Ambachew Kebede

Data curation: Azmeraw Ambachew Kebede, Birhan Tsegaw Taye, Kindu Yinges Wondie, Agumas Eskezia Tiguh, Getachew Azeze Eriku, Muhabaw Shumye Mihret

Formal analysis: Azmeraw Ambachew Kebede, Birhan Tsegaw Taye, Kindu Yinges Wondie, Agumas Eskezia Tiguh, Getachew Azeze Eriku, Muhabaw Shumye Mihret

Investigation: Azmeraw Ambachew Kebede, Muhabaw Shumye Mihret, and Birhan Tsegaw Taye

Methodology: Azmeraw Ambachew Kebede, Birhan Tsegaw Taye, Kindu Yinges Wondie, Agumas Eskezia Eriku, Getachew Azeze Eriku, Muhabaw Shumye Mihret

Validation: Azmeraw Ambachew Kebede, Birhan Tsegaw Taye, Kindu Yinges, Agumas Eskezia Tiguh, Getachew Azeze Eriku, Muhabaw Shumye Mihret

Visualization: Azmeraw Ambachew Kebede, Birhan Tsegaw Taye, Kindu Yinges Wondie, Agumas Eskezia Tiguh, Getachew Azeze Eriku, Muhabaw Shumye Mihret

Writing-original draft: Azmeraw Ambachew Kebede

Writing- review and editing: Azmeraw Ambachew Kebede, Birhan Tsegaw Taye, Kindu Yinges, Agumas Eskezia Tiguh, Getachew Azeze Eriku, Muhabaw Shumye Mihret

Table 1: Socio-demographic characteristics of study participants in Gondar province hospitals, northwest Ethiopia, 2020/2021 (n= 406)

Characteristics	Category	Frequency	Percentage (%)
**Age of participant in year**	≤25	85	20.9
	26-30	247	60.8
	≥31	74	18.2
**Sex of the participant**	Male	272	67
	Female	134	33
**Current marital status**	Single	164	40.4
	Married	242	59.6
**Work experience**	≤2	140	34.5
	3-5	210	51.7
	≥6	56	13.8
**Having smart phone or computer**	Yes	256	63.1
	No	150	36.9
**Ever watching television**	Yes	353	86.9
	No	53	13.1
**Ever reading newspaper**	Yes	207	51
	No	199	49
**Professional category**	Midwifery diploma	119	29.3
	Midwifery degree	243	59.8
	Midwifery master's degree	25	6.2

	Others*	19	4.7
**Monthly income**	<5000 ETB	140	34.4
	5001- 10000 ETB	239	58.9
	>10001 ETB	27	6.7

Note: * GP, Residents, and IESO; ETB, Ethiopia Birr;

Table2: Workplace and professional related characteristics of study participants in Gondar province hospitals, northwest Ethiopia, 2020/2021 (n= 406)

**Characteristics**	**Category**	**Frequency**	**Percentage (%)**
**Facility type**	Primary hospital	214	52.7
	General hospital	32	7.9
	Tertiary hospital	160	39.4
**Facility location**	Urban	223	54.9
	Semi-urban	183	45.1
**Self-rated relation with the nearby manager**	Good	314	77.3
	Poor	92	22.7
**Satisfaction on the profession**	Satisfied	324	79.8
	Unsatisfied	82	20.2
**Availability of internet in the hospital**	Yes	232	57.1
	No	174	42.9
**Working time**	Day	295	72.7
	Night	111	27.3
**Assistant present for the delivery**	Yes	318	78.3
	No	88	21.7
**PNC guidelines in the maternity ward**	Yes	218	53.7

	No	188	46.3
**Received BEmONC training**	Yes	126	31
	No	280	69
**When did you take the training (n= 126)**	Within 2 years	66	52.4
	Before 2 years	60	47.6
**Intention to stay in the profession**	Yes	290	71.4
	No	116	28.6
**Interest to work in the delivery room**	Yes	340	83.7
	No	66	16.3
**Workload load in the ward**	Yes	179	44.1
	No	227	55.9
**Working part-time at private health facility**	Yes	51	12.6
	No	355	87.4
**Regular checkup by the nearby manager**	Yes	287	70.7
	No	119	29.3
**Education while working**	Yes	151	37.2
	No	255	62.8
**Training on essential newborn care**	Yes	156	38.4
	No	250	61.6
**When did you take the essential newborn care training**	Within 2 years	102	65.4
	Before 2 years	54	34.6

Note: PNC, postnatal care; BEmONC, basic emergency obstetric and neonatal care

Table 3: Adherence to immediate postpartum care checklist among health care providers at hospitals in Gondar Province northwest Ethiopia, 2020/2021

Immediate postpartum checklists	Frequency
Sex of neonate recorded	360 (88.7%)
APGAR score assessed	365 (89.9%)
Thermal protection ensured	318 (78.3%)
Oxytocin provided for the woman	388 (95.6%)
Vitamin K given for the neonate	373 (91.9%)
Tetracycline eye ointment given	390 (96.1%)
Weigth of the newborn measured	386 (95.1%)
Uterine massage done	352 (86.7%)
Maternal vital signs checked	318 (78.3%)
Placental completeness checked	317 (78.1%)
Early breastfeeding initiated	363 (89.4%)
Counsel on danger signs and immunization	309 (76.1%)
Counsel on breastfeeding attachment and positioning	321 (79.1)
Head circumference measured	199 (49%)
Length of the newborn measured	198 (48.8%)
Proper documentation done	341 (84%)

Table 4: Factors associated with immediate postpartum care guidelines among healthcare provider's at hospitals in Gondar province, northwest Ethiopia, 2020/2021 (n= 406)

Variables	Category	Adherence to immediate postpartum care		COR (95%CI)	AOR (95%CI)
		Complete	Incomplete		
Intention to stay in their profession	Yes	130	152	2.19 (1.38, 3.47)	1.59 (0.93, 2.71)
	No	34	82	1	1
Marital status	Married	123	119	2.43 (1.6, 3.7)	1.59 (1.15, 3.31)*
	Unmarried	49	115	1	1
Checkup nearby manager for immediate postpartum cares	Yes	138	149	2.32 (1.46, 3.67)	1.11 (0.61, 2.02)
	No	34	85	1	1
Media exposure	Exposed	158	195	2.26 (1.6, 3.7)	0.86 (0.38, 1.98)
	Unexposed	14	39		
Having smartphone or computer	Yes	128	128	2.41 (1.57, 3.69)	1.49 (0.91, 2.44)
	No	44	106	1	1
Satisfaction on their job	Satisfied	149	175	2.84 (1.28, 3.71)	1.19 (06.3, 2.25)
	Unsatisfied	23	59	1	1
Presence of internet connection in the facility	Yes	112	120	1.77 (1.18, 2.66)	0.79(0.47, 1.35)
	No	60	114	1	1
Presence of immediate postpartum care guidelines in the ward	Yes	122	96	3.51 (2.31, 5.34)	2.39 (1.44, 3.98)**
	No	50	138	1	1
Presence of assistant for the delivery	Yes	145	173	1.89 (1.14, 3.34)	1.87 (1.10, 3.33)*
	No	27	61	1	1
Received training on essential	Yes	84	72	2.15 (1.43, 3.23)	0.85 (0.49, 1.46)

newborn care	No	88	162	1	1
Received training on BEmONC	Yes	77	49	3.06 (1.98, 4.73)	2.1 (1.2, 3.6)*
	No	95	185	1	1
Age of healthcare providers in year	≤ 25	31	54	1	1
	26-30	102	145	1.23 (0.74, 2.04)	0.88 (0.49, 1.57)
	≥31	39	35	1.94 (1.03, 3.66)	1.66 (0.68, 4.04)
Experience in year	≤2	47	93	1	1
	3-5	112	98	2.26 (1.45, 3.52)	1.14 (0.66, 1.97)
	≥6	13	43	0.59 (0.29, 1.22)	0.15 (0.06, 0.38)
Average monthly income	<5000 ETB	45	95	1	1
	5001-10000 ETB	114	125	1.92 (1.24, 2.97)	1.2 (0.722, 2.02)
	≥10001 ETB	13	14	1.96 (0.85,4.51)	3.55 (1.30, 9.67)*

Notes: * P≤0.05, **P ≤0.001

Abbreviations: AOR, adjusted odds ratio; BEmONC, basic emergency obstetric and neonatal care; COR, crude odds ratio; CI, confidence interval; ETB, Ethiopia birr; 1, reference category

References

1. WHO Recommendations on Postnatal care of the mother and newborn. WHO. 2013;
2. Mathai Matthews, von Xylander Severin and ZJ. WHO Technical Consultation on Postpartum and Postnatal Care. Dep Mak pregnacy safer. 2010;
3. Beraki GG, Tesfamariam EH, Gebremichael A, Yohannes B. Knowledge on postnatal care among postpartum mothers during discharge in maternity hospitals in Asmara : a cross- sectional study. 2020;8:1–10.
4. Presidential Task Force on Redefining the Postpartum Visit Committe on Obstetrci Practice. Am Coll Obstet Gynecol. 2018;131(728):35–42.
5. Trends in maternal mortality 2000 to 2017: estimates by WHO, UNICEF, UNFPA, World Bank Group and the United Nations Population Division. Geneva: World Health Organization; 2019: Licence: CC BY-NC-SA 3.0 IGO.; 2017.
6. United Nations Inter-agency Group for Child Mortality Estimation (UN IGME), ‘Levels & Trends in Child Mortality: Report 2020, Estimates developed by the United Nations Inter-agency Group for Child Mortality Estimation’, United Nations Children’s Fund, New. 2020;
7. Trends in Maternal Mortality Estimates by WHO, UNICEF, World Bank Group, and the UN Population Division. UNICEF Data: Monitoring the Situation of Children and Women. September 2019.
8. Benova L, Owolabi O, RadovichID E, Wong KLM, Macleod D, LangloisID E V., et al. Provision of postpartum care to women giving birth in health facilities in sub-Saharan Africa : A cross-sectional study using Demographic and Health Survey data from 33 countries. PLOS Med [Internet]. 2019;88:1–19. Available from: http://dx.doi.org/10.1371/journal.pmed.1002943
9. Dlamini BR, Ziyane IS, Gule WP. AIDS & Clinical Research The Quality of Immediate Postnatal Care in Health Facilities in Swaziland : Experience of Postnatal Mothers. J AIDS Clin Res. 2017;8(3).
10. Kinney M V, Kerber KJ, Black RE, Cohen B, Nkrumah F, Coovadia H. Sub-Saharan

Africa ’ s Mothers , Newborns , and Children : Where and Why Do They Die ? PLOS Med. 2010;7(6):1–9.

11. Langlois É V, Miszkurka M, Zunzunegui V, Ghaffar A. Systematic reviews Inequities in postnatal care in low- and middle-income countries : a systematic review and meta-analysis. Bull World Heal Organ 2015;93259–270G [Internet]. 2015;93(January):259–70. Available from: doi: http://dx.doi.org/10.2471/BLT.14.140996

12. Central Statistical Agency. Ethiopia DHS 2000. 2001;1–299. Available from: http://www.dhsprogram.com/pubs/pdf/FR118/FR118.pdf

13. Central Statistical Agency, ORC Macro. Ethiopia Demographic and Health Survey 2005. Heal San Fr [Internet]. 2006;(September):[446]. Available from: http://www.measuredhs.com/pubs/pdf/FR179/FR179[23June2011].pdf

14. Central Statistical Agency [Ethiopia] and ICF International. 2012. Ethiopia Demographic and Health Survey 2011. Addis Ababa, Ethiopia and Calverton, Maryland, USA: Central Statistical Agency and ICF International.

15. Centeral statical agency of Ethiopia and ICF. Ethiopian demographic and health survey. Addis Ababa, Ethiopia, and Rockville, Maryland, USA: CSA and ICF.2016.

16. Ethiopians National Health Care Quality Strategy for 2016-2020 placed Maternal and Child Health as a priority with the ambitious goals to reduce the maternal mortality ratio from 412 to 199 per 100, 1000; to reduce neonatal mortality from 28 to 10 per 100.

17. Figueiredo KMS, Gonçalves GAA, Batista HMT, Akerman M, Pinheiro WR, Nascimento VB. Actions of primary health care professionals to reduce maternal mortality in the Brazilian Northeast. Int J Equity Health. 2018;17(104):1–8.

18. Nove A, Friberg IK, de Bernis L, McConville F, Moran AC, Najjemba M, et al. Potential impact of midwives in preventing and reducing maternal and neonatal mortality and stillbirths: a Lives Saved Tool modelling study. Lancet Glob Heal [Internet]. 2020;9(1):e24–32. Available from: http://dx.doi.org/10.1016/S2214-109X(20)30397-1

19. Sandall J, Soltani H, Gates S, Shennan A, Devane D. Midwife-led continuity models

versus other models of care for childbearing women. Cochrane Database of Systematic Reviews 2016, Issue 4. Art. No.: CD004667. DOI: 10.1002/14651858.CD004667.pub5.

20. Gavine A, MacGillivray S, McConville F, Gandhi M, Renfrew MJ. Pre-service and in-service education and training for maternal and newborn care providers in low- and middle-income countries: An evidence review and gap analysis. Midwifery [Internet]. 2019;78:104–13. Available from: https://doi.org/10.1016/j.midw.2019.08.007

21. Filby A, McConville F, Portela A. What prevents quality midwifery care? A systematic mapping of barriers in low and middle income countries from the provider perspective. PLoS One. 2016;11(5):1–20.

22. Haftu A, Hagos H, Mehari MAB, Brhane G. Health care providers ' adherence to immediate postpartum care guideline and associated factors among women who gave birth in Mekele public teaching hospitals , Tigray. BMC Res Notes [Internet]. 2018;1–5. Available from: https://doi.org/10.1186/s13104-018-3845-0

23. WHO recommendations on maternal health guidelines approved by the WHO guidelines review committee.Geneva: World Health Organization; 2017 (WHO/MCA/17.10). Licence: CC BY-NC-SA 3.0 IGO.

24. Final list of proposed Sustainable Development Goal indicators. Report of the Inter-Agency and Expert Group on Sustainable Development Goal Indicators (E/CN.3/2016/2/Rev.1). 2016; Available from: https://sustainabledevelopment.un.org/content/documents/11803Official-List-of-Proposed-SDG-Indicators.pdf

25. Tegbar Yigzaw, catherine carr, Jelle stekelenburg, Jos van roosmalen, Hannah gibson, Mintwab gelagay azeb admassu. Using task analysis to generate evidence for strengthening midwifery education , practice , and regulation in Ethiopia. Int J Womens Health. 2016;8:181–90.

26. Shen Y, Li Q, Liu X, Xiao S, Yan H. Training and fi nancial intervention for encouraging maternal health service utilization. :1–7.

27. The State of the Ethiopian’s Midwifery based on Ethiopian midwives association

database. 2012;

28. Important new partnership with Ethiopian Midwives Association. July 18, 2016.

29. Otolorin E, Gomez P, Currie S, Thapa K, Dao B. Essential basic and emergency obstetric and newborn care : From education and training to service delivery and quality of care. Int J Gynecol Obstet j. 2015;130.

30. Arba A. Knowledge of Essential Newborn Care and Associated Factors among Nurses and Midwives : A Cross-Sectional Study at Public Health Facilities in Wolaita Zone , Southern Ethiopia , 2019. 2020;2020.

31. Tilahun M, Mengistie B, Egata G, Reda AA. Health workers ' attitudes toward sexual and reproductive health services for unmarried adolescents in Ethiopia. 2012;1–7.

32. Tasneem S, Cagatan AS, Avci MZ, Basustaoglu AC. Job Satisfaction of Health Service Providers Working in a Public Tertiary Care Hospital of Pakistan. 2018;17–27.

Questionnaire

Click here to access/download
Supporting Information
questionnaire on IPPg.docx
