# Supplementary material for: Healthcare provider’s adherence to immediate postpartum care guidelines in Gondar province hospitals, northwest Ethiopia: A multicenter study
Source: PLoS One. 2021 Oct 28;16(10):e0259263. doi: 10.1371/journal.pone.0259263 (PMC8553061; doi:10.1371/journal.pone.0259263)
Supplement: Attachment — Submitted filename: Response to Reviewers.docx [file pone.0259263.s005.docx]

**July 29, 2021**

**Manuscript ID:** PONE-D-21-13480

**Response to Reviewers**

First of all, we would like to acknowledge your constructive comments on and valuable improvements to our paper. We have incorporated all the suggestions and comments with tracked changes in the revised manuscript and a point-by-point response, please see below.

**Response to Editor’s Comments**

| S.NN | Comments | Responses |
| --- | --- | --- |
| 01 | Please ensure that your manuscript meets PLOS ONE's style requirements, including those for file naming | Thank you very much, we have revised the manuscript as per your kind recommendation; PLOS ONE style template. |
| 02 | In your Data Availability statement, you have not specified where the minimal data set underlying the results described in your manuscript can be found. PLOS defines a study's minimal data set as the underlying data used to reach the conclusions drawn in the manuscript and any additional data required to replicate the reported study findings in their entirety. All PLOS journals require that the minimal data set be made fully available. | Thank you, dear editor, we have included the data availability statement and all the required file is included within the paper. |

**Response to Reviewer (s) comments**

**Reviewer #1**

| S.N | Comments | Responses |
| --- | --- | --- |
| 01 | The main sentence is not well corresponded with the sentence in the parenthesis. > Sub-Saharan Africa (SSA) merely accounted for more than two-thirds of the global MM (i.e., 542 maternal deaths per 100, 000 live births) | Thank you, for your insightful comments and deep review. We really appreciate your effort. We tried to relate the sentence and did a reconstruction of sentence in the revised manuscript. |
| 02 | Please define what PPP means. > Thus, about 20 to 44% of these maternal deaths occur during the PPP | Dear reviewer, thank you for your careful reading. Unfortunately, it was missed and corrected in the manuscript. |
| 03 | We need relevant information to understand the fact of 'low healthcare access'. > Ethiopia is one of the SSA countries with the highest maternal and neonatal mortalities and low healthcare access | Thank you, dear reviewer, we tried to elaborate by adding a sentence about the maternal healthcare access, which will make it clear about the description. Further information about maternal and neonatal mortality is described in paragraph four. |
| 04 | Several words - professional, providers, and midwife - are repeatedly appeared in this sentence. Please require reconsiderations. > To reduce maternal and neonatal mortalities on a permanent and sustainable basis, healthcare professionals, especially midwives, play the lion’s share [18] as healthcare providers, in specific midwives, are the frontline care providers for women and newborns | Thank you, dear reviewer, we tried to make it clear and reconcile the ideas. |
| 05 | **METHODS** This sentence has several verbs, so please correct it. > These include medical doctors, midwives, and integrated emergency surgeon officers (IESO) working at hospitals in northwest Ethiopia during the data collection period were included. | Thank you, dear reviewer, for your kind recommendation, we accept that the sentence is insignificant and removed in the revised manuscript. |
| 06 | In the sample size calculation, how do the authors consider cluster effect? Because each facility can be considered as a cluster which affected to practices of the providers in the same facility as the authors discussed in this manuscript. | Thank you, for your insightful concern. You are right, it could possible to consider each health facility as a cluster. However, as far as we can get the total number of healthcare providers and possible to prepare sampling frame, it is better to use a simple random sampling technique and the effect of inter-facility practice will not be problem, as we think. But we really appreciate your way of looking. |
| 07 | Definition of several variables and data processing procedure are not clear. For instance, how the satisfaction was treated only into two categories despite of several questions; is dichotomization of the answer to 'self-rated relation with the nearby boss' sufficient?; criteria to assess 'proper documentation' is not clear; what does 'Presence of assistant for the delivery' mean, who was the assistant, what was the standard role of the assistant; oxytocin provided to women - when and how?; APGAR score at when?; Uterine massage - when and how? etc. Please add sufficient explanation in the main text, or in annex. | We would like to appreciate your viewpoint and way of understanding. The satisfaction was assessed using nine questions and we make it dichotomized to make it clear and easily understandable for policymakers. So, it is included in the operational definition part in the revised manuscript and the dichotomization is based on previous literature. Self-rated relation with the nearby boss is another variable in which we intended to measure what looks like the self-rated relationship of the healthcare providers with their immediate supervisor. Because self-perception towards someone or something else may have great impact which in turn will affect individual’s activity. However, measuring relationship with a single “yes” or “no” question may not be appropriate but we used it for only descriptive purpose since we didn’t able to get composite variables to measure it. Thus, it was not included in the multivariable logistic regression analysis. For the rest variables such as proper documentation, presence of an assistant, oxytocin provision, APGAR score documentation, and uterine massage, we prepared an observation guide to the data collectors on how to ask and document the variables. So, as per your kind recommendation we included it in the revised manuscript as a supporting information and will be posted along with the paper. For your further information, see the revised manuscript in the supporting information section, please. |
| 08 | How the observation was conducted should be described. For instance, just one case of care by a provider was observed, or several observations were used for the assessment | Thank you, dear reviewer, you raised a very essential question. One provider was observed only once and efforts have been made to minimize the Hawthorns effect. More detail is included in the revised manuscript, data collection method and procedures section. |
| 09 | I do not understand what '5%' means. > Before the actual data collection, a pretest was done on 5% of healthcare providers outside of the study area. | Sorry for the inconvenience, it was to mean that pretest was done on 5% of the calculated sample size (i.e. on 22 healthcare providers). We tried to make it clear in the revised manuscript. |
| 10 | **RESULTS**  In the first part of the results section, the number of recruited participants, the number of actual participants, and respondent rate should be described | Thank you, dear reviewer, we tried to correct it as per your kind recommendation and shown with track change in the revised manuscript. |
| 11 | This sentence is not complete. Does it show the proportion of providers who performed full adherence? > The proportion of healthcare provides adhering to IPPCG was 42.4% (95% CI: 37.5, 47.2). | Thank you, dear reviewer, for your attention and careful reading. Yes, it was to mean fully or completely adhering to IPPCG and correct in the revised manuscript. |
| 12 | I recommend the authors to modify Table 3. Please display the items according to the time from the birth up to six hours of postpartum. | Thank you, dear reviewer, fully accepted. Even if most of the cares can be given simultaneously by the primary birth attendant and the assistant person, we tried to modify in the revised manuscript based on the World Health Organization and other organization recommendations- please, kindly take a look in the revised manuscript. |
| 13 | Was there any consideration of collinearity in the multiple logistic regression model? If yes, how was it evaluated and treated? I guess that age and experience should have a positive linear relationship. Media exposure and having PC or mobile is as well. | Thank you, dear reviewer, for your deep review and essential question. Multicollinearity test was done and it was in the acceptable range. That means, the variance inflation factor (VIF) for all variables was less than 10. A single sentence describing this is included in the revised manuscript-see the document, please. |
| 14 | **DISCUSSION**  I am not convinced by this speculation: > The discrepancy might be due to variation in the study population. The study from Mekelle includes nurses and midwives whereas our study incorporates midwives, medical doctors, and IESO. Because the proportion of medical doctors and IESO in this study is less than 5%. The authors did not show relationship between the qualification and the adherence, so the claim is not valid by the data. | Thank you dear reviewer. You are we did not show the prelateship between qualification and adherence. We removed the explanation and rearranged in the revised manuscript. |

**Reviewer #2**

| S.N | Comments | Responses |
| --- | --- | --- |
| 01 | In my opinion the article is very well written and the survey was planned well. Authors clearly indicates the scope of the rules to be followed when caring for the mother and the newborn baby immediately after childbirth. The authors paid attention to the recommendations in the field of prophylaxis and their implementation. | Thank you, dear reviewer, for your appreciation and encouragement. We are grateful to appreciate your scientific contribution and important addition to our manuscript. |
