# Supplementary material for: Healthcare provider’s adherence to immediate postpartum care guidelines in Gondar province hospitals, northwest Ethiopia: A multicenter study
Source: PLoS One. 2021 Oct 28;16(10):e0259263. doi: 10.1371/journal.pone.0259263 (PMC8553061; doi:10.1371/journal.pone.0259263)
Supplement: Attachment — Submitted filename: Response to Reviewers,.docx [file pone.0259263.s006.docx]

**September 6, 2021**

**Title:** Healthcare provider’s adherence to immediate postpartum care guidelines in Gondar province hospitals, northwest Ethiopia: A multicenter study

**Manuscript ID:** PONE-D-21-13480R1

**Response to Reviewer**

First of all, we are grateful to the whole editorial team and the honourable reviewer for your careful attention and thoughtful comments. We believed that with the help of thoughtful comments of the reviewer, the quality of our manuscript has been improved substantially. We have incorporated all the suggestions and comments with tracked changes in the revised manuscript and a point-by-point response, please see below.

Reviewer **#1**

| S.N | Comments | Responses |
| --- | --- | --- |
| 01 | I do not understand why the authors stated 'the effect of inter-facility practice will not be problem' in the response without showing any evidence. This is not right attitude as a peer-review process in a scientific paper. If we see the positive result in this article, it is apparent that setting of each facility affected to the outcome, since 'availability of postpartum care guidelines at the maternity ward' has significant effect. The authors mixed up individual factors (i.e. marital status, income) and facility factors (i.e. presence of internet connection, presence of guidelines) in its analysis. So that individual response cannot be treated individually. In addition, as I have pointed out, the reasons why cluster effect was not considered in the study design should be clearly explained. | Thank you, dear reviewer. First of all, please accept our apologies. Unfortunately, we made a mistake in our description. As you pointed out, it is obvious each health facility can be considered as a cluster and there will be differences in practice. However, we fail to put inter-facility practice since the type of health facility was one independent variable, it is possible to identify in which facility (Tertiary hospital, general hospital, or primary hospital) the practice was lower or higher. Besides, at the time of data collection, we assured the study participants as well as the hospital directors that every information is anonymous and will be kept confidential. In the meantime, if we do the practice of each hospital by listing their names, it may create displeasure. Moreover, at the end of the day, we are expected to submit/disseminate our results to each health facility and clearly communicate with the respective stakeholders on what strengths and weaknesses were encountered from the study. We didn’t use the cluster effect since the entire population of the study are internally homogenous (i.e. health care provider’s level of qualification was almost the same). Above and beyond, we feared using the cluster method because cluster sampling is prone to higher sampling error and the inference about the entire population would be biased than the samples formed using other sampling methods simple random sampling.  Lastly, we put as a limitation, ‘‘this study did not address factors related to the health care system and policies that are designed to plan and provide medical care for people’’. |
| 02 | Regarding the job satisfaction, the referred paper (Lu Y et al.) used eight questions while this article mentioned that there were nine questions. An explanation on this difference is expected. | Thank you, dear reviewer, for your deep and careful reading. It was typing error and we used 8 questions to measure job satisfaction. The questionnaire was repeated by mistake (question number 2 &8). However, the data entered into the SPSS was 8 questions. Please kindly see the questionnaire that we have attached in the initial submission and we did a correction on the revised manuscript. Thank you once again for your genuine contribution. |
| 03 | The authors mentioned that 'one provider was observed only once'. Discussion on its validity considering possible fluctuations in individual practices is expected. | Thank you, we agreed you have provided helpful feedback. However, we have employed this for the following main reasons; (1) First, as a cross-sectional study, answers reflect a single moment in time we could not verify the true characteristics of participants and may not be generalizable over time fluctuations in practice as we acknowledge the limitations.  (2) Repeated participating in observation, the observer can inadvertently influence the other participant’s behavior to lose objectivity and may lead to bias. |
| 04 | Following sentence provides a confusion on mortality, mortality rate, and mortality ratio, since MM and NM were not indicated as ratio nor rate. Please revise them:  Thus, the MM was 871 in 2000 [13], 673 in 2005 [14], 676 in 2011 (15), and 412 per 100,000 live births in 2016 [12]. Besides, neonatal mortality (NM) has dropped from 49 in 2000 to 39 in 2005 [13, 14], and 37 in 2011 to 29 per 1000 live births in 2016 [12, 15]. In Ethiopia, as a country, a lot of works has been done to increase maternal healthcare service utilization with the promise of reducing the maternal mortality ratio from 412 per 100,000 live births to 199, and the neonatal mortality rate from 29 to 10 per 1000 live births by 2020 [16]. | Thank you, it was to mean Maternal mortality ratio and neonatal mortality rate. We did a correction in the revised manuscript. Also, we removed it from the list of abbreviations since it was mentioned only once. |
| 05 | Careless mistakes are found. Significant digits in each table and main texts should be unified. Odds ratio and its CI 1.19 (06.3, 2.25) is an apparent mistake, 0.15 (0.06, 0.38) is not significant? | Thank you for your attentive reading and please accept our apologies for the inconveniences caused. The first was typing error and it was to 0.63.  Also, the last one (0.15; 0.06, 0.38) was totally missed elsewhere in the main text of the manuscript. Comment accepted and corrected, it is included under factor associated and discussed sections of the revised manuscript. |
| 07 | Reference list should be reconsidered to follow a standard of the journal. | Thank you, done. |
