# Supplementary material for: Healthcare provider’s adherence to immediate postpartum care guidelines in Gondar province hospitals, northwest Ethiopia: A multicenter study
Source: PLoS One. 2021 Oct 28;16(10):e0259263. doi: 10.1371/journal.pone.0259263 (PMC8553061; doi:10.1371/journal.pone.0259263)
Supplement: Attachment — Submitted filename: Response to reviewers..docx [file pone.0259263.s007.docx]

**October 8, 2021**

**Manuscript ID:** PONE-D-21-13480R2

**Response to Reviewers**

First of all, we would like to acknowledge your constructive comments on and valuable improvements to our paper. We have incorporated the suggestions and comments with tracked changes in the revised manuscript and a point-by-point response, please see below.

**Response to Editor (journal requirements) Comments**

| S.NN | Comments | Responses |
| --- | --- | --- |
| 01 | Please review your reference list to ensure that it is complete and correct.  If you have cited papers that have been retracted, please include the rationale for doing so in the manuscript text, or remove these references and replace them with relevant current references. | Thank you, we thoroughly review the references and there is no retracted paper cited. |

**Response to Reviewer(s) comments**

**Reviewer #1**

| S.N | Comments | Responses |
| --- | --- | --- |
| 01 | I would appreciate it if the authors would state counter-arguments on the following issue. The authors explained why they have to keep information on hospital names confidential in several sentences. But this does not make a sense. Because my previous comment was why the authors do not take cluster effect into account in the analysis. This can be performed without indicating each hospital and participant. The authors explained that 'the entire population of the study are internally homogenous (i.e. health care provider’s level of qualification was almost the same)'; however, this statement is still arbitrary, since no data shows the homogeneity of the participants. In addition, as I have pointed out, the analysis mixed individual and facility factors as the independent variables. One possible solution would be using 'multilevel logistic regression model' instead of binary one. | Dear reviewer, we are grateful for your effort, commitment, timely feedback, and incredible way of looking for the improvement of our manuscript. You did a great contribution to this manuscript many times and inspire us to read more. Coming to the specific concerns, even if there is no sure evidence for the homogeneity of the population, evidence supports that individuals who are drawn from a hospital, school, or a classroom tend to have more homogeneity as compared to those drawn from a population which is large in size (https://doi.org/10.1371/journal.  pone.0225427). However, it does not mean that they are always similar. As you pointed out, the cluster effect might have an influence across institutions on the outcome variable. As a solution, performing multilevel logistic regression has been suggested. Dear reviewer, you are right, performing multilevel logistic regression will be best to minimize cluster effects on hierarchical and cluster based data. In the present study, participants were treated at the individual level and there was no clustering-based on the level or category of health facility. Participants were taken at random irrespective of the type of health facility they are working in during the data collection period. To satisfy the requirements of hierarchical data nature, we would have assessed particular characteristics of each category of health facility with their respective cluster number. To proceed with the multi-level analysis, well-defined clusters are needed and considered as an independent variable. That is why we did not consider multilevel analysis since our data lacks that and other assumptions. By considering the hospitals as a cluster, we tried to compute the intra-cluster correlation coefficient (ICC) between adherence to IPPCG and facility type and were not indicative for multilevel analysis. Thus, The ICC was 0.02 meaning that about 2% of the variation in the outcome variable is attributed to institution-level factors. The ICC cut-off point to proceed with multilevel analysis is controversial, in which some say 5% (<https://doi.org/10.1080/00220973.2014.952397>) and others say 10%. From this, we can understand that as ICC increases (approaches to 1) the institution level factors has a strong effect on the outcome variable and should solely be addressed by multi-level analysis. On the opposite, if it approaches 0 effects of institution-level factors is low. However, this might not be a reliable finding since we didn’t have well-defined clustered based data. Given your great concern in consideration and to make it clear for readers and policymakers, we have acknowledged it as a limitation being analyzing individual and facility-level factors in mixed will have an effect-please see the revised manuscript. Thank you once again for your important addition to our manuscript. |
